# Assessing the treatment response of lateral elbow tendinopathy using time-dependent ultrasonography, Doppler imaging, and elastography

**DOI:** 10.1186/s13244-024-01695-8

**Published:** 2024-05-11

**Authors:** David Tobaly, Patrice Tétreault, Guy Cloutier, Manon Choinière, Philippe Grondin, Véronique Freire, Anne-Sophie Julien, Nathalie J. Bureau

**Affiliations:** 1 Department of Radiology, St Mary’s Hospital Center, 3830 Lacombe Avenue, Montreal, QC H3T 1M5 Canada; 2 Department of Orthopedics, Centre hospitalier de l’Université de Montréal (CHUM), 1000 rue Saint-Denis, Montreal, QC H2X 0C1 Canada; 3 Research Center, Centre hospitalier de l’Université de Montréal (CHUM), 900 rue Saint-Denis, Montreal, QC H2X 0A9 Canada; 4 Department of Anesthesiology and Pain Medicine, Université de Montréal, C.P. 6128, succursale Centre-ville, Montreal, QC H3C 3J7 Canada; 5 Department of Radiology, Centre hospitalier de l’Université de Montréal (CHUM), 1000 rue Saint-Denis, Montreal, QC H2X 0C1 Canada; 6 Department of Mathematics and Statistic, Université Laval, 1045 avenue de la Médecine, Quebec City, QC G1V 0A6 Canada

**Keywords:** Tennis elbow, Tendinopathy, Elastography, Ultrasonography (Doppler), Dry needling

## Abstract

**Objective:**

To investigate the structural alterations, neovascularity, and elasticity of tendons and the relationship between elasticity and the Patient Rated Tennis Elbow Evaluation score after undergoing US-guided fenestration or surgery in patients with chronic lateral elbow tendinopathy.

**Methods:**

Participants from the per-protocol population of a randomized trial conducted between October 2016 and June 2020 were included. The surgery and fenestration groups included 24 (mean age, 50 ± 7 years [standard deviation], 10 men) and 29 (47 ± 8 years, 18 men) participants, respectively. Ultrasound exams were performed at baseline, 6 months, and 12 months. Statistical analyses included linear mixed effects and generalized equation estimation models.

**Results:**

Fenestration had no significant impact on tendon thickness (*p* = 0.46). Conversely, surgery significantly increased tendon thickness at 6 months (*p* < 0.0001) and remained elevated at 12 months (*p* = 0.04). Tendon echostructure exhibited a group effect (*p* = 0.03), indicating a higher proportion of pathological scores in the surgery group post-intervention compared to the fenestration group. Both groups showed a similar reduction in neovascularity from 6 to 12 months postintervention (*p* = 0.006). Shear-wave velocity increased in the fenestration group at 6 months (*p* = 0.04), while the surgery group experienced a nonsignificant decrease at 6 months, with some improvement at 12 months (*p* = 0.08). Changes in shear-wave velocity did not correlate with clinical outcome.

**Conclusions:**

Fenestration and surgery reduced tendon neovascularity over time. Unlike surgery, fenestration did not impact tendon size while improving tendon echostructure and elasticity.

**Critical relevance statement:**

Fenestration and surgery equally alleviated symptoms and decreased tendon neovascularity in lateral elbow tendinopathy; however, fenestration did not alter tendon thickness and improved echostructure and shear-wave velocity, suggesting shear-wave velocity’s potential for quantitatively monitoring tendon elasticity during healing.

**Key Points:**

- Reliable markers for monitoring healing response and informing treatment protocols in elbow tendinopathy are lacking.
- Fenestration and surgery reduced tendon neovascularity, while fenestration improved tendon echostructure and shear-wave velocity.
- Shear-wave velocity may provide quantitative measures to monitor tendon elasticity in response to treatment.

**Graphical Abstract:**

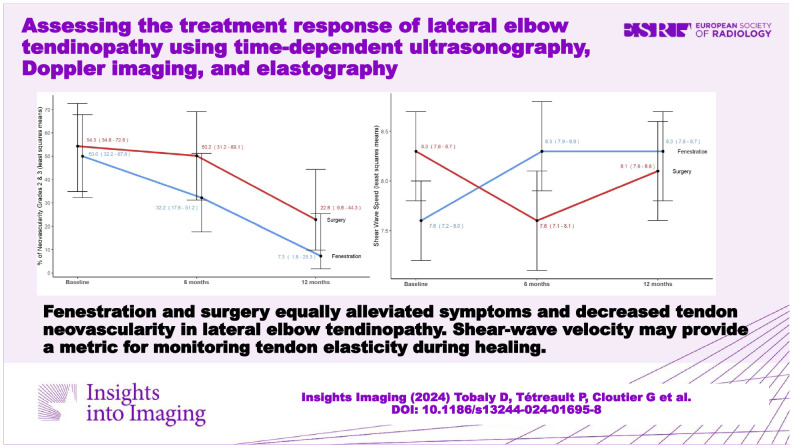

## Introduction

Lateral elbow tendinopathy (LET) [1], or tennis elbow, is a common overuse disorder in primary care, occupational, and sports medicine [2]. The optimal management and timing for surgery in refractory cases are unclear due to a lack of reliable markers to monitor healing response [3, 4].

Imaging studies investigating the structural response of human tendons to treatment are limited. One systematic review found no correlation between clinical outcomes and observable changes in tendon size, neovascularity, and structural abnormalities [5]. One study reported reduced tendon size and neovascularity after corticosteroid injections compared to platelet-rich plasma or saline injections [6]. Another study found that tendon fenestration combined with platelet-rich plasma injection increased tendon thickness and neovascularity in the short and medium term but decreased in the long term [7]. Conversely, fenestration with lidocaine injection resulted in a linear decrease in both parameters. A study on rotator cuff repair showed a decrease in supraspinatus tendon thickness and neovascularity [8].

Ultrasonography and power Doppler imaging characterize LET, but their subjective qualitative assessment of structural changes in the tendon poses a challenge [9, 10]. To address this, new imaging techniques have emerged to provide quantitative information on the mechanical properties of tissues. Among them, shear-wave elastography (SWE) is a reliable and repeatable method to measure the elasticity in phantoms [11], muscles [12], and tendons [13, 14]. This imaging modality can distinguish between symptomatic and asymptomatic tendons by quantifying pathological softening in diseased tendons [15]. Studies have also demonstrated that SWE is sensitive to tendon stiffness during nonsurgical treatment of various tendinopathies, including some cases of LET [16] and surgical repair of Achilles tendon ruptures [17]. However, more research is necessary to define how SWE can monitor LET treatment response.

The first aim of this study was to investigate the time-dependent changes in B-mode US structure, power Doppler neovascularity, and shear-wave velocity (SWV) in the common extensor tendon and radial collateral ligament complex (CET-RCL) following US-guided tendon fenestration (dry needling) or open-release surgery in patients with chronic LET. The second aim was to evaluate whether changes in the severity of symptoms, using the patient-rated tennis elbow evaluation (PRTEE) score, were associated with tendon SWV.

## Subjects and methods

This prospective ancillary study, employing a randomization ratio of 1:1, addresses the secondary objectives of the clinical trial that compared the therapeutic efficacy of US-guided tendon fenestration with open-release surgery in 64 patients with chronic LET [18]. The Institutional Ethics Committee approved the study (CE 15.327), and all participants signed informed consent. The study was registered on Clinicaltrials.gov (NCT02710682), and the study protocol was previously published [19].

### Study population

We report on the study participants who strictly adhered to the protocol and received the allocated intervention in a per-protocol approach. The surgery group included 24 participants (10 [42%] men; mean age 50 ± 7 [standard deviation] years; range 30–61), while the fenestration group included 29 participants (18 [62%] men; 47 ± 8 years; range 33–59). The participants were enrolled in the study and were followed for 12 months between October 2016 and June 2020. The mean duration of symptoms was 19 ± 13 months (range 6–60) and 24 ± 23 months (range 7–120) in the surgery and fenestration groups, respectively.

The inclusion criteria were strict, requiring participants to be between 25 and 67 years old, with unilateral LET refractory to conservative management for at least 6 months. The diagnosis was made by one of two fellowship-trained orthopedic surgeons (PT, PG;18 and 13 years of experience), based on a lateral elbow pain score on resisted dorsiflexion of the wrist, middle finger, or both ≥ 4/10 on a numerical rating scale where 0 = no pain and 10 = worst pain imaginable. The exclusion criteria included a CET-RCL tear > 50% of the tendon surface identified at baseline US examination, a history of elbow surgery or fracture, corticosteroid injections received in the last three months before enrolment, and treatment with autologous blood or platelet-rich plasma injections.

The primary clinical outcome measure was the total PRTEE score at 6 months following the intervention. A reduction of 11/100 points from the baseline on the PRTEE questionnaire, corresponding to clinically “much better” or “completely recovered” [20], was considered a successful treatment in the statistical analyses. PRTEE scores were assessed 6 weeks, 3, 6 months, and 12 months postprocedure.

### Interventions

One of two fellowship-trained musculoskeletal radiologists (N.J.B., V.F.) with 23 and 10 years of experience conducted the US-guided tendon fenestration on patients in a supine position using a standardized technique [18, 19]. The procedure followed an aseptic technique and started by administering 3 mL of 1% lidocaine to anesthetize the skin and subcutaneous tissues. A 22 G needle was then used for US-guided tendon fenestration, passing the needle approximately 20–30 times along the tendon’s long axis to soften the tendinosis area.

The two orthopedists used a standardized open-release surgery technique, which involved incising the skin, reclining the extensor carpi radialis longus (ECRL) tendon, excising the pathological tissue of the extensor carpi radialis brevis tendon, suturing back the ECRL tendon, and ending with skin closure [18, 19].

### Ultrasonography

The participants received a standardized US imaging examination at three-time points: baseline, 6 months, and 12 months after the procedure. One radiologist (NJB) performed the exam using an Acuson S3000 US scanner (Siemens Medical Solutions, Mountain View, CA, USA). Ultrasonography and power Doppler imaging were conducted with a 14L5SP or 14L5 MHz transducer, while SWE was performed with a 9L4 MHz transducer, which was the only linear transducer with SWE capability available on the scanner. The CET-RCL on the lateral aspect of the elbow was scanned in the long axis while participants sat with their elbow flexed at approximately 70° and their pronated forearm resting on the examination table. We used a thick coat of acoustic gel during SWE, and minimal pressure was applied to the transducer. The scanner measured a maximum SWV of 10 m/s, and quality and velocity parametric maps were recorded.

### Ultrasonography, power Doppler imaging, and SWV assessments

Table 1 outlines the grading schemes for the imaging features. The radiologist prospectively assessed and graded the B-mode US, power Doppler, and SWV parameters without reviewing prior imaging data. The scar found on the elbow of participants in the surgery group made it impossible to blind the radiologist to group allocation at the 6- and 12-month follow-up examinations.

**Table 1:** Ultrasound, power Doppler imaging, and SWE grading schemes

Imaging features	Measuring units or grading scores
Tendon thickness	cm
Tendon echostructure	Grade 0: normal
	Grade 1: hypoechogenicity < 30%
	Grade 2: hypoechogenicity 30–70%
	Grade 3: hypoechogenicity > 70% or anechoic clefts
	Grade 4: full-thickness tear or complete tendon detachment
Neovascularity	Grade 0: no pixel
	Grade 1: a few pixels
	Grade 2: $\le$ 50% of the tendon surface
	Grade 3: $>$ 50% of the tendon surface
Enthesophytes	Absent
	Present
Calcifications	Absent
	Present
Tendon elasticity	Mean SWV (m/s)

All imaging features were assessed in the long tendon axis. The tendon thickness was measured at the base of the humeral epicondyle. The SWV is the mean of three equidistant ROI positioned within the tendon from the apex to the base of the epicondyle. *cm* centimeters, *m/s* meter/second

**Fig. 1:**
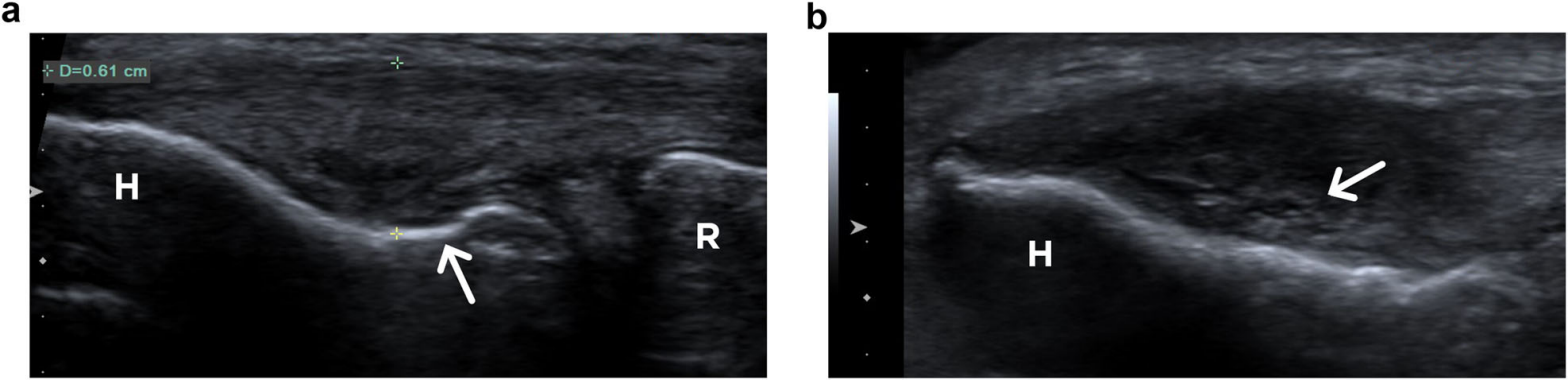
Ultrasound of the right elbow in a 49-year-old man with chronic lateral tendinopathy. **a** The thickness of the common extensor tendon and radial collateral ligament complex (between the cursors) is measured on a long-axis US, showing the minor groove (arrow) at the base of the lateral epicondyle. **b** The entire tendon was scanned to assess its echostructure. On a more anterior image, the tendon is thickened and hypoechoic and shows anechoic clefts (arrow) consistent with an echostructure grade 3. H, humeral epicondyle; R, radial head

**Fig. 2:**
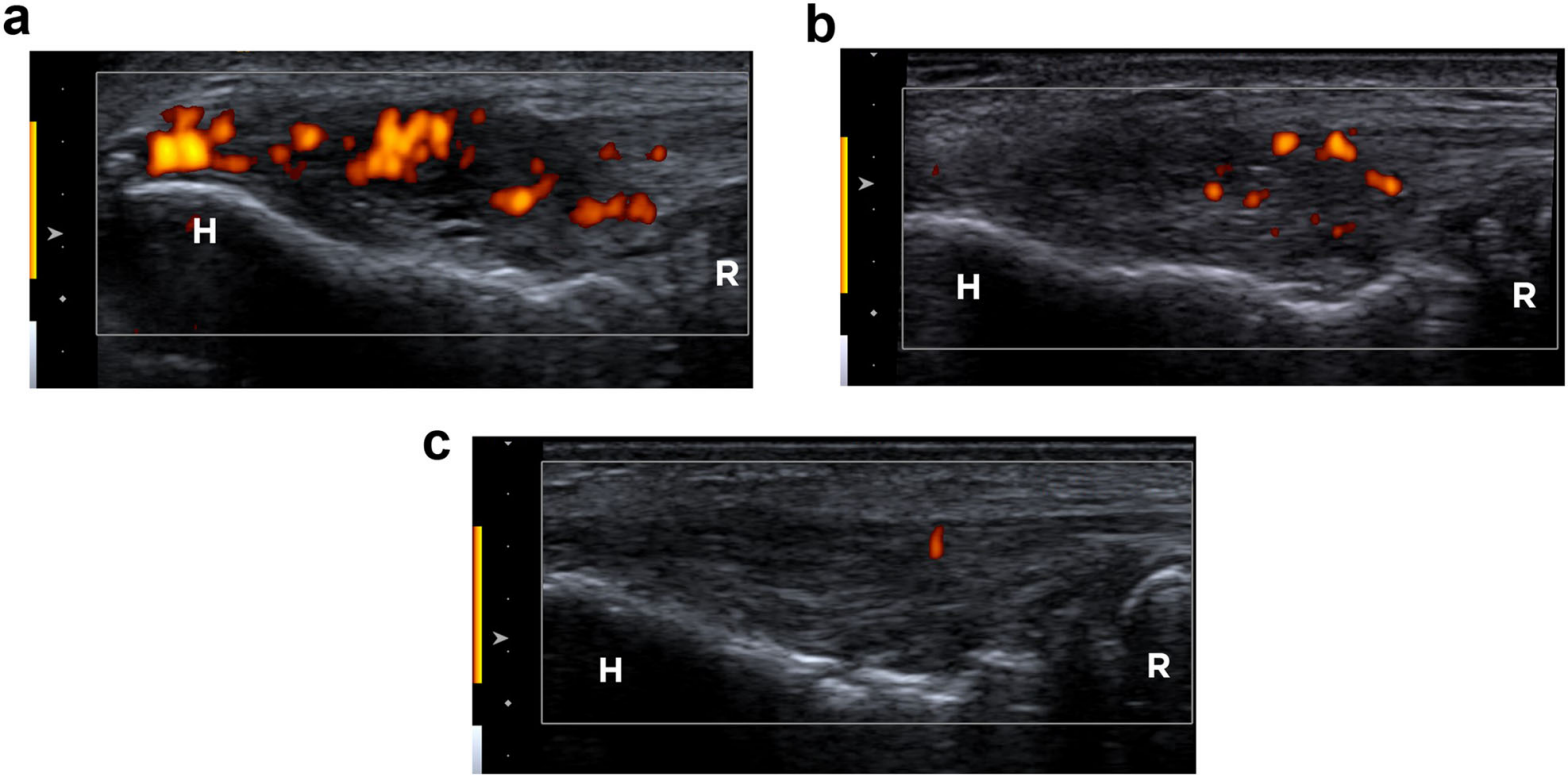
Power Doppler imaging of the right elbow in the same 49-year-old man with chronic lateral tendinopathy. The entire tendon was scanned, capturing the area with the highest neovessel density. **a** Baseline long-axis US with Power Doppler imaging reveals pixels of Doppler signal covering around 50% of the tendon surface, indicating Grade 2 neovascularity. **b** Following tendon fenestration treatment, the 6-month follow-up long-axis US displays partial neovessel regression, with a few pixels of Doppler signal indicating Grade 1 neovascularity. **c** The 12-month follow-up long-axis US exhibits a nearly complete resolution of the Doppler signal (Grade 1 neovascularity)

The thickness of the CET-RCL was measured on the long-axis US image showing the minor groove at the base of the lateral epicondyle. (Fig. 1) The lateral aspect of the elbow was scanned in the anteroposterior direction to examine the entire tendon and to assess its echostructure, the presence or absence of enthesophytes, and tendon calcifications [21]. These features were graded using ordinal or dichotomous scales. The maximum density of neovessels within the CET-RCL was assessed on power Doppler imaging in the long axis using standardized settings (pulse repetition frequency of 977 and gain adjusted at a level just below random noise) and was graded on an ordinal scale (Fig. 2).

**Fig. 3:**
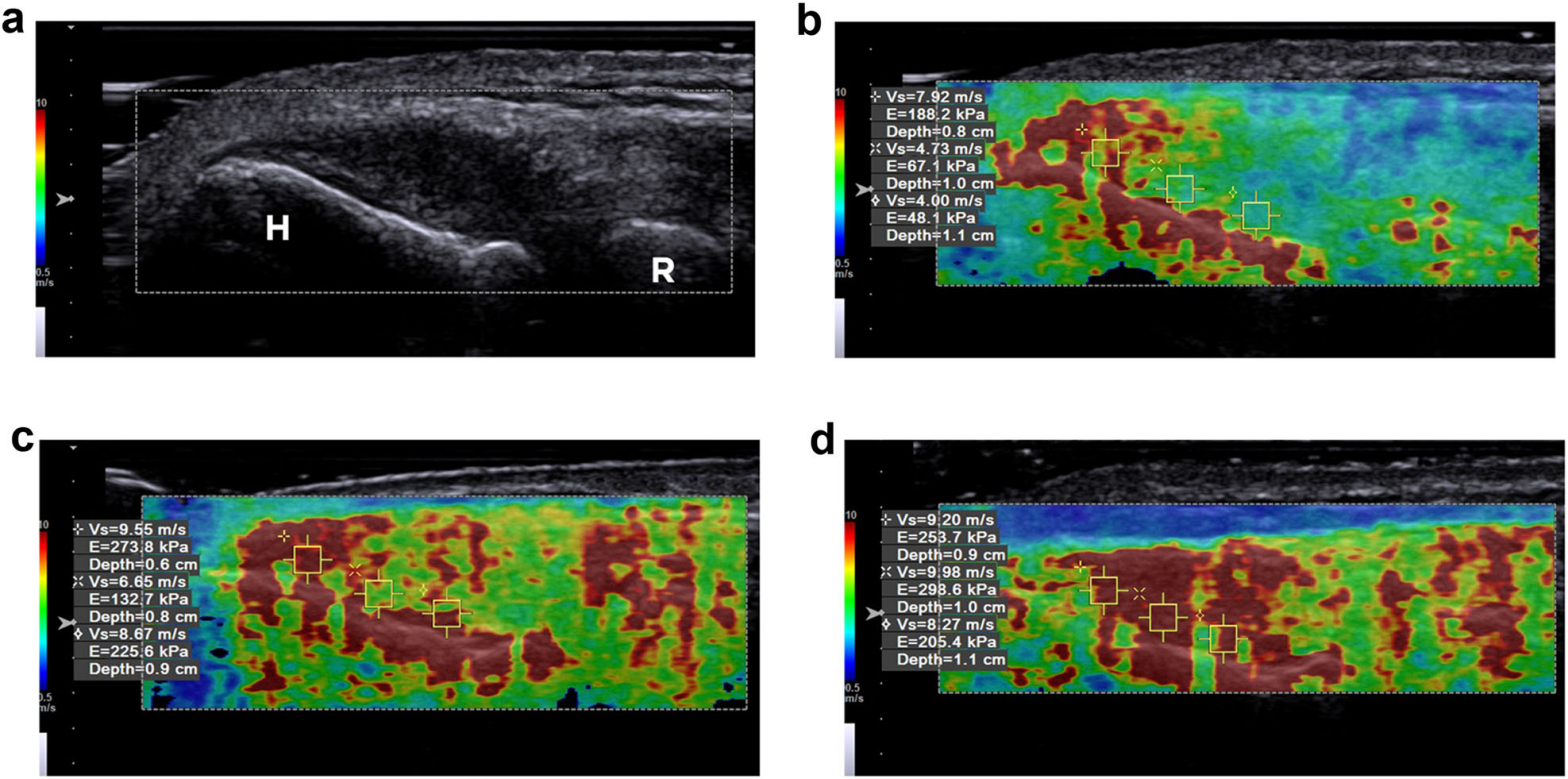
SWE of the right elbow in the same 49-year-old man with chronic lateral tendinopathy. **a** Baseline long-axis SWE of the common extensor tendon and radial collateral ligament complex shows the ROI. **b** Corresponding VTIQ parametric velocity map with three equidistant ROI placed within the tendon, aligned from the apex to the base of the epicondyle to measure the local SWVs. The mean SWV was 5.55 m/s. **c** At the 6-month follow-up exam, the mean SWV was 8.29 m/s. **d** At the 12-month follow-up exam, the mean SWV was 9.15 m/s

Using Siemens’s virtual touch tissue imaging and quantification (VTIQ) software, we assessed SWV on a long-axis image depicting the minor groove at the base of the lateral humeral epicondyle. The transducer was positioned parallel to the humeral cortex, with the incident US beam adjusted perpendicular to the cortex. The clear hyperechoic definition of the cortical bone confirmed this. A 2D shear-wave quality map was initially used to show the CET-RCL complex’s signal-to-noise ratio. The color coding used was green for good quality, yellow for marginal, and red for poor. Once a quality map showed a good signal-to-noise ratio over the CET-RCL complex, the SWV mode was activated. This generated a 2D color map of SWV distribution within the tendon. Three equidistant 1.5 × 1.5 mm regions of interest (ROI) were placed in the CET-RCL complex to capture regional variations in tissue stiffness. These ROIs were aligned from the apex to the base of the epicondyle, and the mean of the three SWV measures was retained for analysis. If SWV exceeded the 10 m/s limit of the Siemens scanner, we assigned a value of 10 m/s to the ROI (Fig. 3).

### Statistical analysis

Descriptive statistics were used to characterize the imaging findings in each group at baseline. A linear mixed-effects model was employed to analyze the continuous outcome variables, such as tendon thickness and mean SWV. The model incorporated treatment, time, and the interaction between treatment and time as fixed effects, while individual patient-specific random intercepts were also considered. Meanwhile, tendon echostructure and neovascularity, which were classified into normal (Grades 0 and 1) and pathological grades (Grades 2, 3, 4 for echostructure; grades 2, 3 for neovascularity), were analyzed with a generalized equation estimation model with a logit link. Additionally, we applied a Bonferroni correction to account for multiple comparisons. Furthermore, Pearson’s correlation was employed to test the correlation between changes in PRTEE score and changes in SWV, considering six sets of variables, including absolute and relative changes in scores from 0 to 6 months, 6 months to 12 months, and 0 months to 12 months, for the whole cohort and per group. Finally, we compared the mean SWV between successful and unsuccessful treatments at 6 and 12 months using an ANOVA adjusted for the group effect. One of the authors (A.S.J.) performed the statistical analyses using SAS software version 9.4 (SAS Institute Inc.).

## Results

### Clinical outcome

At baseline, the mean PRTEE score was 56.8 ± 16.4 (24.0–87.0) in the surgery group and 53.4 ± 16.7 (8.0–81.0) in the fenestration group. As previously reported in this patient cohort [18], the proportion of successful treatment at 6 months was equivalent between the surgery group (20 of 24) and the fenestration group (24 of 29), at 83% (95% confidence interval [CI] 63–95% and CI 64–94%, respectively; *p* = 1.00].

### Ultrasonography

**Fig. 4:**
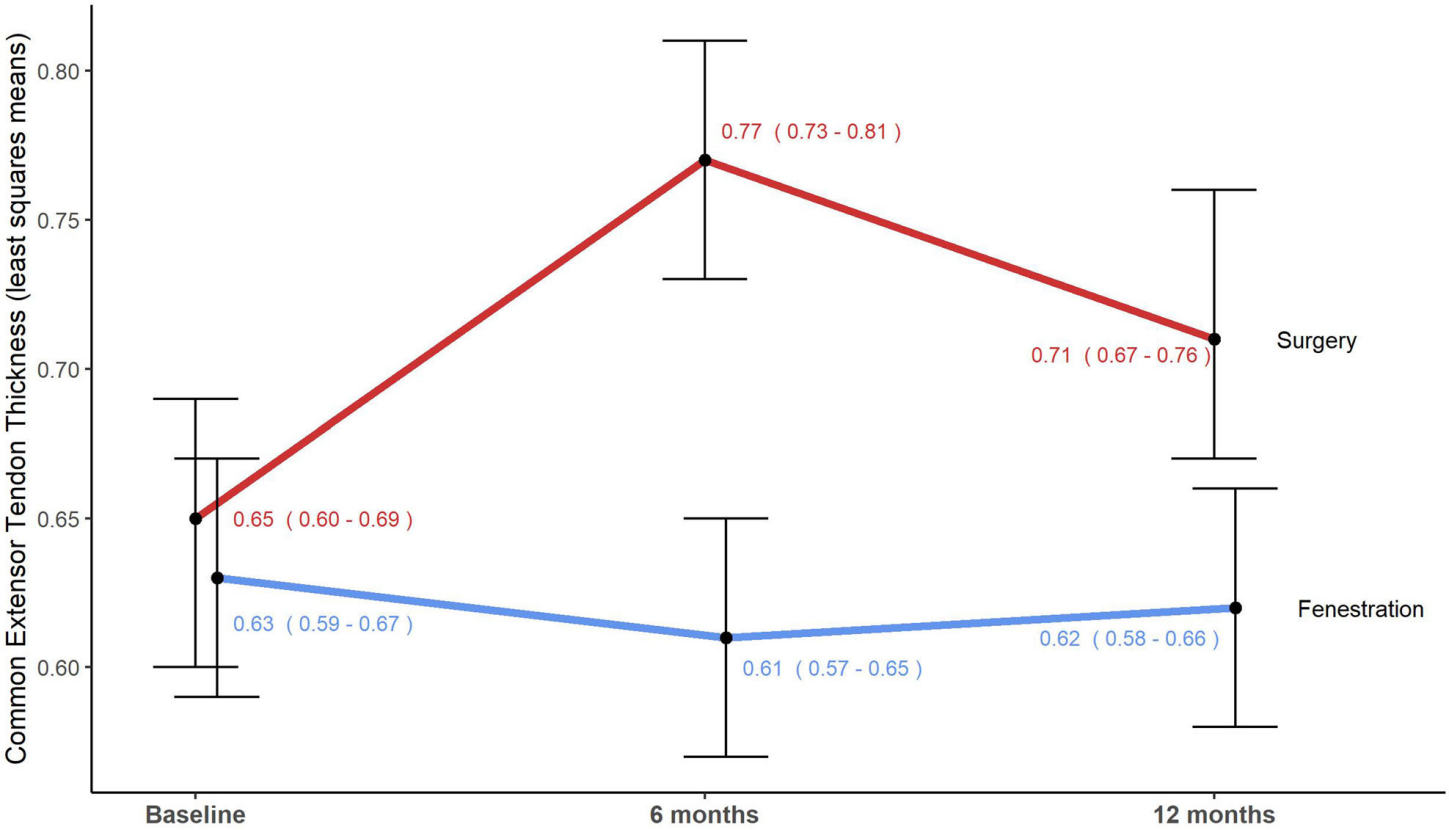
Tendon thickness over time in the same 49-year-old man with chronic lateral tendinopathy. This graph presents the mean tendon thickness over time, measured in centimetres, with their corresponding CI intervals in parentheses and displayed by the bars. Surgery significantly increased tendon thickness, whereas fenestration did not alter it significantly. Statistically significant differences between intervention groups were observed at 6 months (*p* < 0.0001) and 12 months (*p* = 0.003)

The baseline imaging findings are presented in Table 2. There were no changes in the presence or absence of epicondylar enthesophytes and tendon calcifications after either intervention. However, there was a significant treatment-by-time interaction observed in the tendon thickness scores (*F*_(2, 96)_ = 11.99; *p* < 0.0001). Specifically, the fenestration treatment did not alter tendon thickness (*p* = 0.46). Conversely, there was an increase in tendon thickness for the surgery group at 6 months (*p* < 0.0001), persisting at 12 months compared to baseline (*p* = 0.04), despite an improvement between 6 and 12 months (*p* = 0.004) (Fig. 4).

**Table 2:** Baseline imaging findings in the fenestration and surgery groups

Imaging findings	Surgery (*N* = 24)	Fenestration (*N* = 28*)
Tendon thickness (cm)	0.65 ± 0.09 (0.50–0.87)	0.63 ± 0.11 (0.45–1.00)
Tendon echostructure		
Grade 0	0 (0.00)	0 (0.00)
Grade 1	5 (20.83)	5 (17.24)
Grade 2	7 (29.17)	7 (24.14)
Grade 3	12 (50.0)	16 (55.17)
Grade 4	0 (0.00)	0 (0.00)
Neovascularity		
Grade 0	2 (8.33)	4 (13.79)
Grade 1	9 (37.50)	10 (34.48)
Grade 2	12 (50.00)	12 (41.38)
Grade 3	1 (4.17)	2 (6.90)
Enthesophytes		
Absent	1 (4.17)	5 (17.24)
Present	23 (95.83)	23 (79.31)
Calcifications		
Absent	13 (54.17)	15 (51.72)
Present	11 (45.83)	13 (44.83)
Tendon elasticity (m/s)	8.28 ± 1.13 (5.67–9.87)	7.58 ± 1.39 (5.28–9.83)

*N*, number. Continuous data are presented as mean ± standard deviation (range), while categorical data are presented as frequency (percentage)

* Baseline data from one participant in the Fenestration group is missing due to a technical failure of the US scanner at the time of the exam

**Fig. 5:**
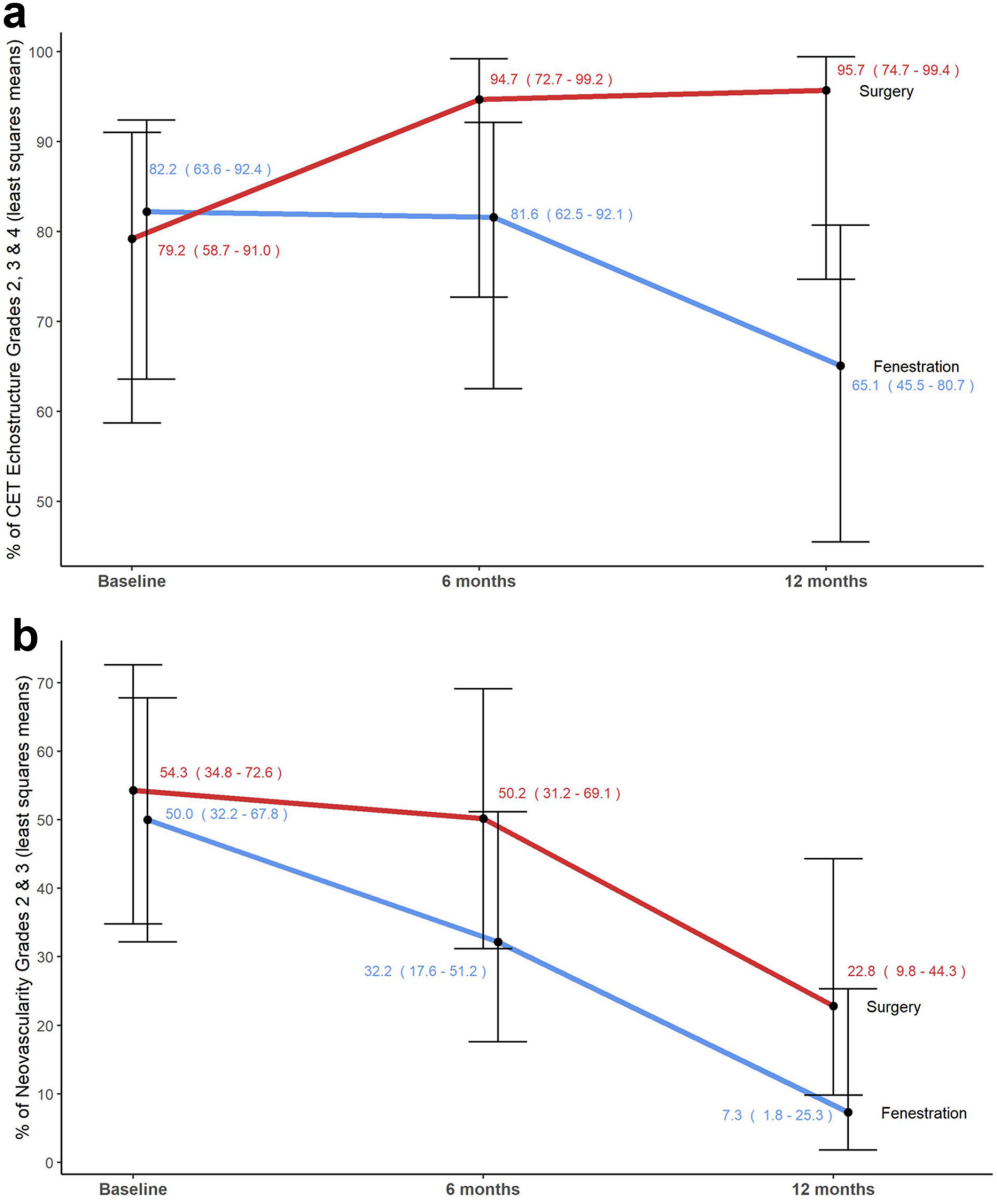
Tendon echostructure and neovascularity pathological scores over time in the same 49-year-old man with chronic lateral tendinopathy. **a** This graph presents percentages of pathological tendon echostructure scores (grades 2, 3, and 4) over time, with CI intervals in parentheses and represented by bars. The surgery group exhibited more structural alterations persisting over time than the fenestration group (*p* = 0.03), while the fenestration group showed potential long-term improvement. **b** The graph displays percentages for pathological neovascularity scores (grades 2 and 3) over time, with CI intervals in parentheses and represented by bars. Both interventions significantly reduced neovessel density between 6 and 12 months postintervention (*p* = 0.006). Although the fenestration technique appeared to achieve faster and greater reduction, there was no difference between intervention groups (interaction *p* = 0.46)

The proportion of tendon echostructure pathological scores over time showed a significant group effect (*p* = 0.03), indicating that the surgery group had a higher proportion of pathological scores than the fenestration group for the study duration (Fig. 5).

### Power Doppler imaging

No significant treatment-by-time interaction was observed in neovascularity scores (*p* = 0.46). Both groups exhibited a significant reduction in the proportion of neovascularity pathological scores from 6 to 12 months post-intervention (*p* = 0.006) (Fig. 5).

### Shear-wave elastography

**Fig. 6:**
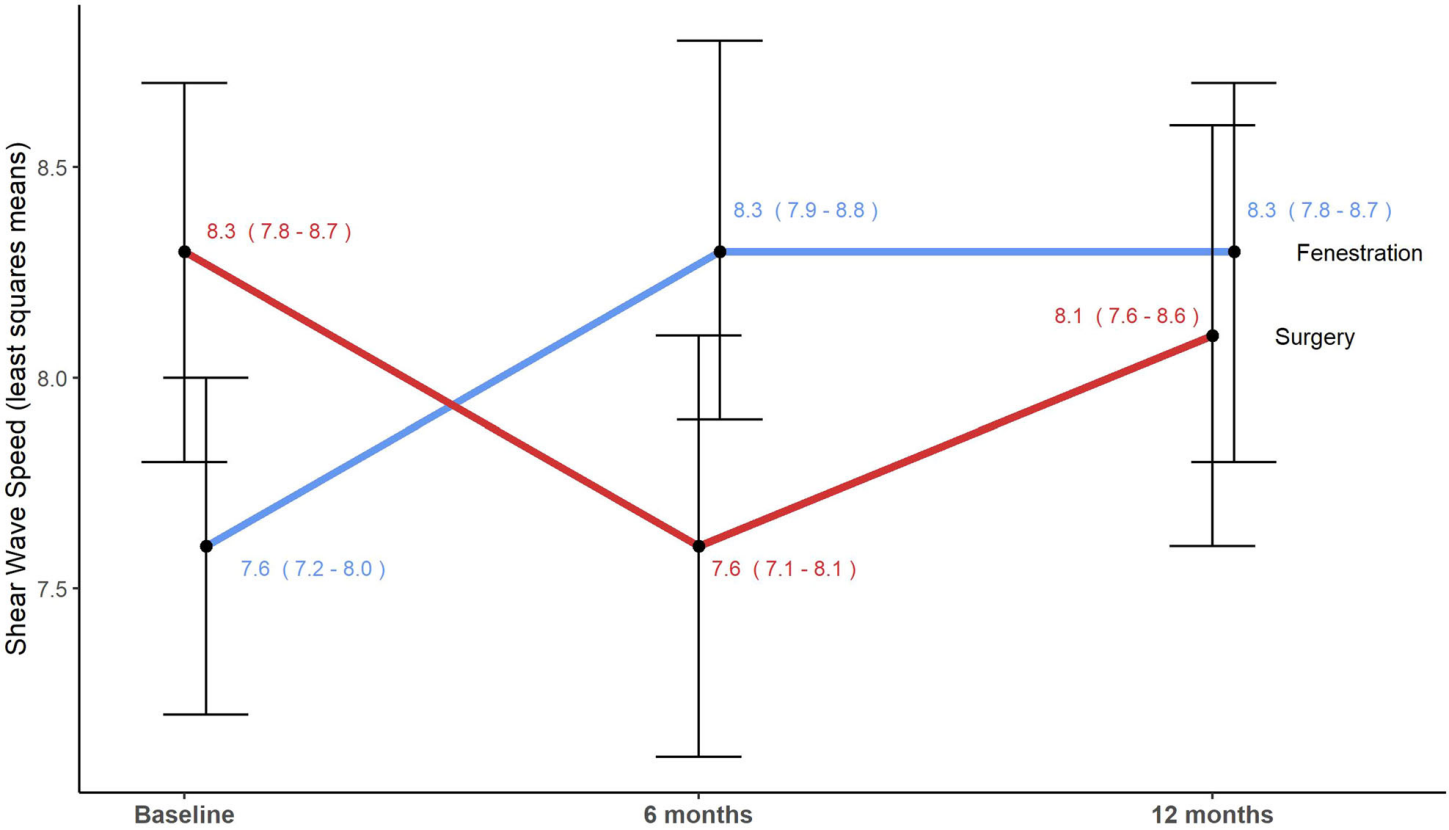
Tendon SWV over time in the same 49-year-old man with chronic lateral tendinopathy. The graph illustrates the mean SWV over time in meters per second, with CI intervals in parentheses and displayed by the bars. Tendon fenestration significantly increased tendon SWV (*p* = 0.03), peaking 6 months postintervention. Conversely, SWV tended to decrease in the open-surgery group at 6 months, with slight improvement at 12 months, but these changes did not reach statistical significance (*p* = 0.08). The group difference was significant at 6 months (*p* = 0.03) but not at 12 months (*p* = 0.56)

This analysis showed a significant interaction between treatment and time in the SWV scores (F_(2, 93)_ = 5.27; *p* = 0.007). Specifically, the fenestration group displayed an increase in tendon SWV at 6 months compared to baseline (*p* = 0.04), which remained constant at 12 months. However, tendon SWV decreased in the surgery group at 6 months, with some improvement at 12 months, although these changes were not statistically significant (*p* = 0.08). At 6 months, there was a significant difference in tendon SWV between the two groups (*p* = 0.03). These findings are presented in Fig. 6.

### Correlation between interval changes in SWV and clinical outcome

There was a moderate correlation between the relative change in SWV and PRTEE score between 6 and 12 months after fenestration (*r* = −0.395, *n* = 26, *p* = 0.046). Additionally, there was a weak correlation between the absolute change in SWV between 6 and 12 months and the absolute change in the PRTEE score between 0 and 6 months when considering the fenestration group and the surgery group together (*r* = −0.296, *n* = 48, *p* = 0.041). In both cases, an increase in SWV was associated with a decrease in PRTEE scores. There were no significant differences in mean SWV between the successful and failed treatments at 6 (*p* = 0.53) and 12 (*p* = 0.78) months.

## Discussion

Tendinopathies commonly exhibit the following histopathological changes: collagen disorganization, cellular density variations, increased fibroblast activity, neovascularity, and extracellular matrix myxoid and hyaline changes [22]. These lead to structural (thickening, hypoechogenicity, neovascularity, tears, calcifications, and enthesophytes) and mechanical (softening) abnormalities that can be detected with imaging techniques to inform care management [23].

Our study revealed that fenestration and surgery equally reduced tendon neovascularity in patients with LET, showing significant changes at 6–12 months postintervention. These results align with prior research on percutaneous therapies and surgery for tendinopathies [7, 8, 24].

However, our study found that fenestration was superior to surgery for maintaining or improving tendon structure. Surgery thickened tendons without enhancing echostructure, while fenestration maintained the tendon’s size and improved echostructure at 6–12 months. This discrepancy may stem from the different techniques used. Surgery ablates immature reparative tissue and repairs the tendon interface through a skin incision [25], while fenestration induces an acute inflammatory response and promotes healing and regeneration percutaneously [26].

Recently, Riggin et al [27] conducted research supporting the proposed healing mechanism of the fenestration procedure. Using US-guided fenestration on normal rat supraspinatus tendons, they compared mild needling (three passes), moderate needling (nine passes), and no needling in a control group. Assessments at 1- and 6-week intervals included Doppler imaging, histological and immunohistochemical analysis, cross-sectional measurement, and mechanical testing (percent relaxation; maximum load, stiffness, and stress; elasticity modulus). Results indicated no permanent tendon damage from needling. Both needling groups initially showed increased blood flow, type III collagen deposition, and inflammation, returning to baseline values by the 6-week. The moderate group exhibited increased tendon size at one week, returning to baseline at 6 weeks, while mild needling did not affect size. Both groups demonstrated decreased stiffness and modulus at one week, with only the mild group fully recovering at 6 weeks. Modulus did not return to baseline in the moderate group. These findings raise questions about the optimal fenestration extent and required time for tendon recovery. The moderate needling group may have fully recovered beyond the 6 weeks covered by the study.

Similarly, our study observed that fenestration elevated tendon SWV in patients with LET over the medium and long term, indicating increased tendon stiffness. In contrast, surgery tended to reduce tendon SWV in the medium term and did not show improvement compared to baseline in the long term. These findings concur with those from animal studies on surgically injured tendons, showing an initial decline and partial recovery of mechanical properties over time [28, 29].

We found a moderate to weak correlation between changes in SWV and clinical outcome, underscoring the need for cautious result interpretation. Increased SWV correlated with decreased PRTEE score, signifying improved clinical outcome. Long-term SWV changes (6–12 months postintervention) exhibited a stronger association with clinical outcome than changes within the first 6 months, suggesting that enhanced tendon stiffness becomes noticeable in the long term. This aligns with the lengthy physiological tendon healing process involving inflammation, repair, and remodeling spanning several months to a year. In the remodeling phase, starting 1–2 months after injury and extending over a year, collagen I synthesis occurs and contributes significantly to increased tendon stiffness [30]. Despite these observations, the absence of statistically significant differences in SWV between successful and failed treatments suggests that factors beyond elasticity influence clinical recovery.

We acknowledge some limitations. First, the sample size was relatively small, which may limit the generalizability of the findings. However, this randomized trial employed rigorous methods and statistical analyses to ensure the reliability and validity of the results. While generalizing to a larger population may be limited, the findings provide valuable evidence for future research. Second, blinded to the patient’s clinical outcome but not to the intervention groups, one radiologist performed the US examinations and imaging parameter assessments. However, the US exams were conducted over three years at 6-month intervals. This extended duration reduced recall bias and provided a representative approach to clinical practice. Third, the Acuson S3000 scanner had a maximum SWV of 10 m/s. Presently, some scanners can assess speeds over 16 m/s. However, only 0.2% (10/477) of our SWV measurements exceeded the 10 m/s limit, unlikely to have significantly impacted our results. A higher-frequency elastography-capable transducer could have been employed for SWV measurement in the CET-RCL complex. However, it is important to note that the spatial resolution of 2D-SWE is inversely related to transducer frequency, and as transducer frequency increases, shear wave propagation diminishes in deeper tissues due to US absorption [31, 32]. Furthermore, Dillman et al’s research [11] demonstrated the reliability and comparability of SWV measurements using the Acuson S3000/9L4 transducer/VTIQ elastography method (the method used in this study) to the Aixplorer (Supersonic Imaging)/SL 10-2 and SL15-4 transducer method at shallow depths (1.0, 2.5, 4.0 cm). Therefore, our selected approach was suitable for accurately evaluating SWV in the CET-RCL complex. We recognize that reflection artifacts off the lateral epicondyle may have affected SWV assessments within the CET-RCL complex. In this longitudinal study, we prioritized a standardized SWV assessment method to ensure reproducible measurements. Additionally, analyzing the delta change of SWV measurements between follow-ups minimized the potential impact of reflection artifacts on our results. We did not assess the inter-reader agreement for SWV, US, and power Doppler imaging parameters. However, in a previous study, we assessed inter-reader agreement for US and power Doppler imaging features of the CET-RCL using the same grading schemes [21]. Our findings revealed an almost perfect inter-reader agreement for maximum tendon thickness scores (intraclass correlation coefficient, 0.84), and perfect (*k* = 1.00) and moderate (*k* = 0.47) inter-reader agreement for power Doppler and echostructure scores, respectively.

In conclusion, while fenestration and surgery equally alleviated clinical symptoms and reduced tendon neovascularity in LET, fenestration exhibited additional benefits by improving tendon structural and mechanical properties. Shear-wave velocity may provide measurements to monitor tendon elasticity during healing. Even though no definitive correlation was observed between SWV changes and clinical outcome, this study underscores the importance of long-term imaging follow-up when monitoring tendon healing response.

## Data Availability

Upon request, the corresponding author can provide access to datasets analyzed in this study.

## Abbreviations

CET-RCL: Common extensor tendon and radial collateral ligament complex
CI: 95% confidence interval
ECRL: Extensor carpi radialis longus
LET: Lateral elbow tendinopathy
PRTEE: Patient rated tennis elbow evaluation
ROI: Region of interest
SWE: Shear-wave elastography
SWV: Shear-wave velocity
VTIQ: Virtual touch tissue imaging and quantification
